# Drought-Induced Regulatory Cascades and Their Effects on the Nutritional Quality of Developing Potato Tubers

**DOI:** 10.3390/genes11080864

**Published:** 2020-07-30

**Authors:** Letitia Da Ros, Raed Elferjani, Raju Soolanayakanahally, Sateesh Kagale, Shankar Pahari, Manoj Kulkarni, Jazeem Wahab, Benoit Bizimungu

**Affiliations:** 1 Faculty of Forestry, University of British Columbia, Vancouver, BC V6T1Z4, Canada; lmdaros@mail.ubc.ca; 2 National Research Council Canada, Saskatoon, SK S7N0W9, Canada; sateesh.kagale@nrc-cnrc.gc.ca (S.K.); ultimatekmanoj@gmail.com (M.K.); 3 Saskatoon Research and Development Centre, Agriculture and Agri-Food Canada, Saskatoon, SK S7N0X2, Canada; cosmos.gob@gmail.com (R.E.); shankar.pahari@canada.ca (S.P.); jazeem.wahab@canada.ca (J.W.);; 4 Fredericton Research and Development Centre, Agriculture and Agri-Food Canada, Fredericton, NB E3B4Z7, Canada; benoit.bizimungu@canada.ca

**Keywords:** crop genetics, *Solanum tuberosum*, abiotic stress, phenylpropanoids, essential amino acid, transcriptome, small RNA, comparative genomics, nutrition

## Abstract

Competition for scarce water resources and the continued effects of global warming exacerbate current constraints on potato crop production. While plants’ response to drought in above-ground tissues has been well documented, the regulatory cascades and subsequent nutritive changes in developing tubers have been largely unexplored. Using the commercial Canadian cultivar “Vigor”, plants were subjected to a gradual drought treatment under high tunnels causing a 4 °C increase in the canopy temperature. Tubers were sampled for RNAseq and metabolite analysis. Approximately 2600 genes and 3898 transcripts were differentially expressed by at least 4-fold in drought-stressed potato tubers, with 75% and 69% being down-regulated, respectively. A further 229 small RNAs were implicated in gene regulation during drought. Expression of several small RNA clusters negatively correlated with expression of their six target patatin genes, suggesting involvement in the regulation of storage proteins during drought. The comparison of protein homologues between *Solanum tuberosum* L. and *Arabidopsis thaliana* L. indicated that down-regulated genes were associated with phenylpropanoid and carotenoid biosynthesis. As is indicative of reduced flow through the phenylpropanoid pathway, phenylalanine accumulated in drought-stressed tubers. This suggests that there may be nutritive implications to drought stress occurring during the potato tuber bulking phase in sensitive cultivars.

## 1. Introduction

Potatoes are the fourth most consumed food crop worldwide and are an efficient source of energy, vitamins and minerals in the human diet [1]. High consumption rates and moderate concentrations of dietary antioxidants have led potatoes to be the third-largest source of total phenolics in the American diet [2]. Diets rich in phenolics have been implicated in the prevention of an array of degenerative diseases and concentrations of these compounds vary greatly based on the cultivar, highlighting the potential for the targeted breeding of potato (*S. tuberosum* L.) to enhance global human health [3]. In potato tubers, the primary polyphenol is chlorogenic acid with the remaining components comprised of carotenoids, anthocyanins, and flavonoids. While the phenolic content of tubers is largely genotype-dependent, the phenolic profiles are driven by the environmental conditions present during growth, tuber bulking, and throughout storage. In general, the production and accumulation of these compounds are favored at lower temperatures, with potatoes grown in warm, dry regions producing lower amounts of phenolics [4]. Environmental parameters could be manipulated to manage the concentrations of desired phytonutrients [4,5,6].

Substrate entry into the general phenylpropanoid pathway in eudicots is driven by phenylalanine ammonia-lyase (PAL), an enzyme that regulates the deamination of phenylalanine to yield the cinnamic acid from which monolignols, flavonoids, and anthocyanins are produced [7]. PAL activity responds to a variety of developmental and environmental cues, with transcriptional regulation occurring by way of MYB, LIM, and KNOX transcription factors [8]. Furthermore, independent MYB transcription factors play a prominent role in the regulation of anthocyanin and flavonoid biosynthesis genes such as flavonol synthase (FLS), flavanone 3-hydroxylase (F3H) and flavonoid 3′–hydroxylase (F3′H), while expression of genes such as dihydroflavonol reductase (DFR) require MYB transcriptional complexes. MicroRNAs (miRNAs) and small interfering RNAs (siRNAs) have notable functions in the regulation of phenylpropanoid biosynthesis in eudicots through the targeting of MYBs, the most notable being miR858, miR828 and TAS4 [7,9]. Causal miRNA and the target MYB transcription factors have been previously identified in potato leaves under drought [10], however, the regulatory cascades present in potato tubers are still unknown.

In addition to phenolics, concentrations of available essential amino acids affect the nutritional value of potato tubers. Up to 50% of the amino acids in tubers are aspartic acid and glutamic acid, with the remaining portion made up of leucine, valine, alanine, lysine, and arginine with the total protein nutritional value of a potato being comparable to an egg white [11]. Genotypes for improving protein quality have been identified among non-traditional potato cultivars [12]. Essential amino acids function both as substrates for secondary metabolism and as a source of energy [13]. As a result, concentrations fluctuate in response to environmental stressors due to concurrent protein degradation and *de novo* synthesis. The transcriptional regulation of amino acid biosynthesis is highly complex and their function during stress response is still unclear [13].

Drought stress is one of the primary concerns for potato production given the projected increases in aridity. Potato is adapted to temperate climates with optimal tuber growth occurring at temperatures between 15 and 20 °C. Temperatures above this range, coupled with periodic drought, have resulted in reduced yields and increased incidences of tuber physiological defects [14]. Symptoms of drought in potato include reduced leaf size, increased chlorophyll content, reduced stomatal conductance, and wilting. However, rooting depth and plant recovery have been shown as the best indicators of plant susceptibility to drought [15,16]. Through comparisons between genotypes with differing tolerance to drought, novel potato drought-responsive genes and transcript markers for drought tolerance in potato leaves have been identified [17,18]. Gene responses in developing potato tubers to drought conditions are not well-documented outside of targeted metabolic pathways [3].

This study aimed at identifying drought-associated changes in developing potato tubers (i.e., tuber bulking phase) and their impacts on nutritional quality. The assessment of transcriptional changes in genes with metabolic functions and quantification of amino acid concentrations aims to guide production and harvest practices when optimizing the nutritional value of the crop. Analysis of small RNAs seeks to identify components of the drought regulatory cascade in potato tubers which, to our knowledge, has yet to be explored.

## 2. Materials and Methods

### 2.1. Experimental Design and Plant Growth Conditions

**Figure 1:**
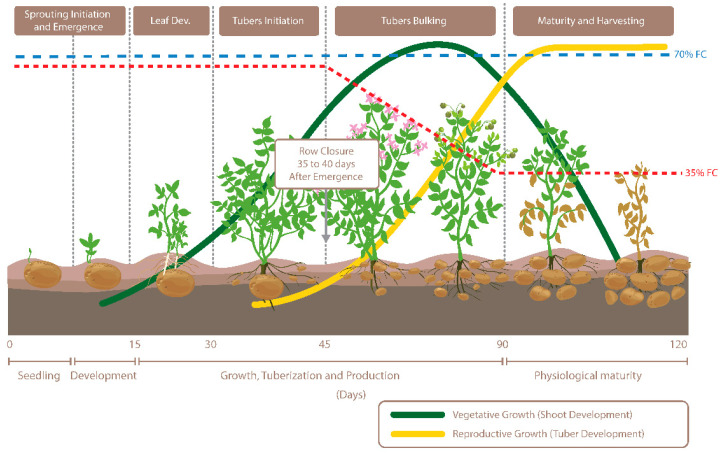
Visualization of potato growth stages and in-season soil moisture trends when soil moisture was maintained at 35% and 70% field capacity (FC).

This study was conducted during the summer of 2017 at the Agriculture and Agri-Food Canada’s Canada-Saskatchewan Irrigation Diversification Centre in Outlook (51°29′ N, 107°03′ W, 541 m), Saskatchewan. The cultivar “Vigor”, a cross between “Agria” and “Wischip” from the Agriculture and Agri-Food Canada Lethbridge Research and Development Centre, was evaluated for its performance under soil moisture stress during the tuber bulking phase. Prominent characteristics of the cultivar are its yellow-fleshed tubers and pigmented (red-violet) flowers. Plants were grown under optimum soil moisture conditions at 70% field capacity (FC) and restricted soil moisture conditions at 35% FC under two high tunnels using drip irrigation. Treatments were imposed at the start of the tuber bulking phase for gradual exposure to drought stress, thereby mimicking natural field conditions (Figure 1). High tunnels were opened from all sides but covered with plastic film on top to mimic the open field condition while preventing rainfall (Supplementary Figure S1A). Plots were laid out in a randomized complete block design containing four replicates with guard rows on either side. Each plot consisted of 12 hills. The two end hills were considered as guard hills for yield estimation purposes. Seed pieces were spaced 1 m between-rows and 20 cm within-rows and were planted on 30 May 2017. The crop was raised using standard management practices (i.e., fertility, irrigation, pest control, etc.). Pre-plant basal fertilizer included urea (46-0-0), mono-ammonium phosphate (11-52-0), and potash (0-0-60). Two applications of ammonium sulphate (21.5-0-0-4) were given at 4 and 7 weeks after planting. Soil moisture was monitored using Watermark sensors (Supplementary Figure S1B). Plots were harvested on 2 October 2017 and graded according to commercial-grade standards.

### 2.2. Physiological Measurements

Physiological measurements were taken at the end of the tuber bulking phase (90th day after planting) with readings recorded consistently between 11:30 a.m. and 12:30 p.m. Canopy temperature was assessed with an infra-red thermal imaging camera (FLIR T530, FLIR Systems. Wilsonville, OR, USA), and leaflet chlorophyll content (CCI) was recorded using a chlorophyll content meter (CCM-200- Apogee Instruments, Logan, UT, USA). Quantum yield of dark-adapted leaflets (*Fv/Fm*) was measured using the portable fluorometer FluorPen FP 100 (PSI, DRASO Czech Republic). Detachable clips were used to dark-adapt the leaflets for 20 min, and *Fv/Fm* was measured on the adaxial surfaces of the top 3rd and 4th leaflet of each sampled plant (three plants per replication).

### 2.3. Amino Acid Profiling and Abscisic Acid Content

In both high tunnels from each replication, a tuber was collected from the middle of the plot on 13 September 2017 (106 DAP). The tubers were washed in running water, followed by distilled water, cut into cubes while evading the skin and immediately frozen in liquid nitrogen. Samples were stored at −80 °C until further use.

Amino acids were extracted from 10 mg of ground freeze-dried tissue following Inaba et al. (1994) [19] with some modifications. Briefly, 1 mL of 80% (*v/v*) ethanol solution (40 °C) was added to each sample, shaken for 30 min at 40 °C and the supernatant was recovered by centrifugation (4000 rpm for 10 min) at 4 °C. The pellets were re-extracted under the same conditions with an additional 500 µL of 80% (*v/v*) ethanol (40 °C). The supernatants were combined and stored at −20 °C until further use. Amino acids were derivatized following Waters AccQTag Reagent Kit (Waters, Milford, MA, USA; [20]). Briefly, a 10 µL aliquot of sample was mixed with 70 µL borate buffer and 20 µL AccQFluor reagent which was reconstituted in acetonitrile. AccQFluor reagent was reconstituted as follows: 1 mL of AccQFluor reagent diluent was transferred to a vial containing AccQFluor reagent powder and vortexed for 10 s before heating at 55 °C for a maximum of 10 min or until dissolved. The derivatized mixture was transferred to an autosampler vial and incubated at 55 °C for 10 min. High-performance liquid chromatography (HPLC) was conducted, as described in Waters AccQTag’s chemistry package instruction manual, with samples separated on a Waters amino acid column −3.9 × 150 mm and quantified at an excitation wavelength of 285 nm and an emission wavelength of 320 nm using a 2475 scanning fluorescence detector (Waters, Milford, MA, USA). The column was set at 37 °C with a 5 µL injection volume. Waters AccQTag buffer (100 mL AccQTag Buffer concentrate +1000 mL deionized water), acetonitrile, and deionized water were used as mobile phases A, B, and C, respectively.

Abscisic acid content was determined following Yan et al., (2016) [21]. Samples were centrifuged to remove debris, and the pellet was washed twice. The supernatant was evaporated in a SpeedVac, and reconstituted in 1 mL of 1% (*v/v*) acetic acid. Abscisic acid (ABA) was purified by solid-phase extraction using Oasis HLB, MCX, and WAX cartridge columns (Waters, Milford, MA, USA). The solvent was removed under vacuum and subjected to LC-ESI-MS/MS analysis (Agilent 6410 TripleQuad LC/MS system). An LC (Agilent 1200 series) equipped with a 50 × 2.1 mm, 1.8-μm Zorbax SB-Phenyl column (Agilent Technologies, Santa Clara, CA, USA) was used with a binary solvent system comprised of 0.01% (*v/v*) acetic acid in water (solvent A) and 0.05% (*v/v*) acetic acid in acetonitrile (solvent B). Separations were performed using a gradient of increasing acetonitrile content at a flow rate of 0.2 mL min^−1^. The gradient was increased linearly from 3% B to 50% B over 15 min. The retention time of ABA was 14 min.

### 2.4. Transcriptome and Small RNA Sequencing

Total RNA was extracted from 100 mg of tuber tissue partitioned from the sample taken for metabolite analysis using the RNeasy plant mini kit (Qiagen, Hilden, Germany). RNA quality and concentration were verified using an Agilent Bioanalyzer (Agilent Technologies, Santa Clara, CA, USA). TruSeq RNA and small RNA sequencing libraries were constructed following the standard preparation guide (Illumina, San Diego, CA, USA). All eight RNA samples (four replicates of each treatment) were multiplexed in a lane of a flow cell and paired-end sequencing (125 cycles) was performed using an Illumina HiSeq 2500. Similarly, for small RNA sequencing, all 8 samples were multiplexed in a lane of a flow cell and single-end sequencing was carried out on Illumina HiSeq 2500.

### 2.5. RNA and Small RNA Read Mapping and Analysis

Before read mapping and expression quantification, all RNA reads were filtered using Trimmomatic (version 0.36; [22]) by (i) removing adapter sequences, (ii) trimming leading and trailing low-quality sequences, (iii) removing sequences when the average quality per base dropped below 15 within a 4-base wide sliding window and (iv) keeping only those pairs where both reads were longer than 75 bp. Clean reads were aligned to the potato reference genome (SolTub_3.0, EnsemblPlants) with STAR (v2.5.2b) and isoform expression was quantified with the RSEM (v1.3.3) algorithm [23]. The expected read counts generated by the RSEM algorithm were rounded off and fed into DESeq2.

The quality of small RNA sequencing reads was assessed using the FASTQC program (v0.11.8; [24]). Reads were quality-filtered and adapter-trimmed using cutadapt (v2.8; [25]). The alignment of filtered reads to the potato reference genome (SolTub_3.0, EnsemblPlants) and annotation and quantification of small RNAs was carried out using ShortStack (v3.8.5; [26]). psRNATarget [27] was used to predict the miRNA and small RNA target genes.

### 2.6. Differential RNA Expression Analysis

Raw read counts obtained from RNAseq were normalized and assessed for differential expression using the Statistical Software “R” version 3.6.0 and the package DESeq2 [28,29]. Log2 fold change threshold of 2 and a 5% false discovery rate (FDR) were used as cut-off values for continuing to the annotation step. R scripts are available on Bioconductor (https://bioconductor.org/) from the package developers and were adapted for the data presented in this paper. The same technique was repeated for the discovery of differentially expressed small RNA, with target gene identification done using psRNATarget [27]. Gene annotations for *S. tuberosum* and *A. thaliana* were obtained from the Ensembl Plants database (http://plants.ensembl.org). *Arabidopsis* homologs with >50% identity to the original potato gene were input into the online DAVID Bioinformatics Resources version 6.8 (https://david.ncifcrf.gov/) for functional annotation clustering and KEGG pathway mapping analyses.

## 3. Results

### 3.1. Physiological Response

A high degree of variability existed within the four control and four treatment plots for the agronomic and physiological traits measured during this study (Table 1). Both crop yield and tuber number per plot were not found to be significantly different from one another, although differences in plot averages were observed. Calculated values from spectral measurements, such as CCI and *Fv*/*Fm*, showed no significant differences. However, canopy temperatures measured in plots maintained at 35% FC were 3.9 °C higher than control plots with soil moisture maintained at 70% FC (Table 1). ABA concentrations in the well-watered control and water-deficit conditions were 43.9 and 55.4 ng gDW^−1^, respectively. Variation was high among the control plants, resulting in no significant differences between treatments.

### 3.2. Tuber Amino Acid Fluctuations in Response to Soil Moisture Deficit

**Figure 2:**
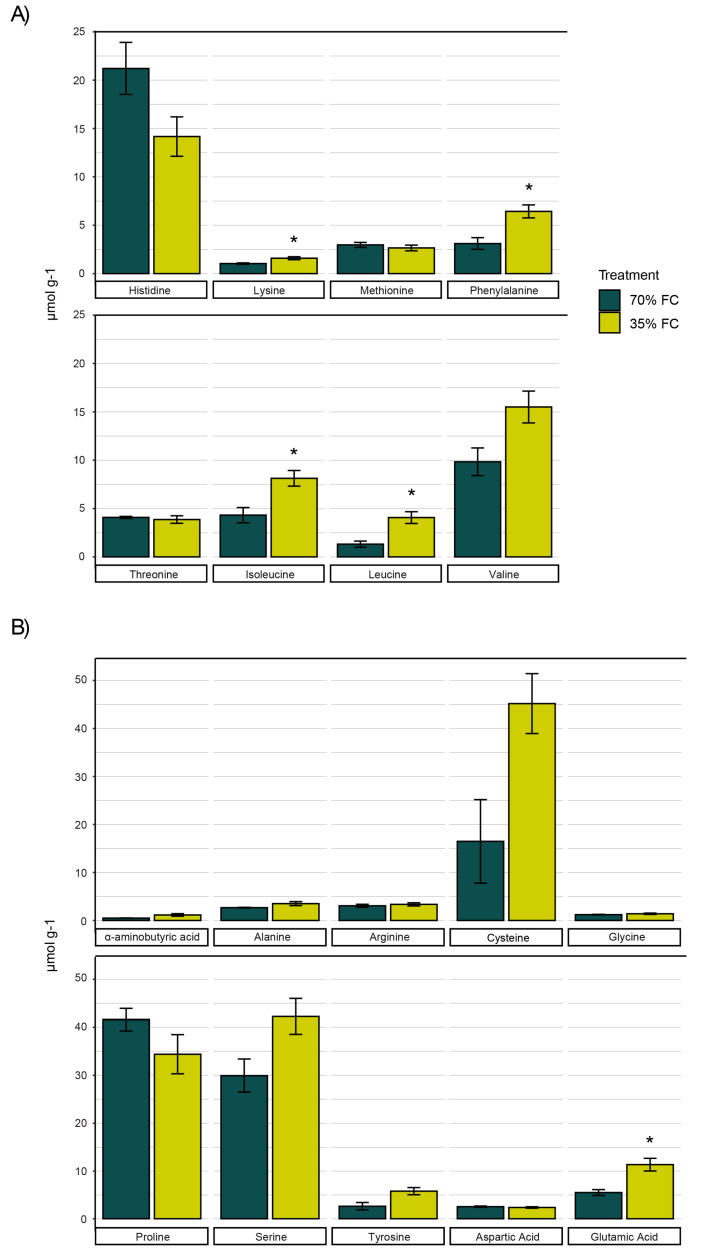
Concentrations of essential (**A**) and non-essential (**B**) amino acids in potato tubers (*n* = 4) sampled during the tuber bulking phase and the associated SEM (*p* < 0.05) when subjected to 70% and 35% field capacity. * significance (*p* < 0.05) between treatments.

Of the eight essential amino acids, lysine, phenylalanine, isoleucine, and leucine were found to be more abundant in drought-stressed tubers. The largest differences were observed in the concentrations of leucine, phenylalanine, and isoleucine which increased by 3×, 2×, and 1.9×, respectively (Figure 2A). Quantities of branched-chain amino acids, a group that includes leucine, isoleucine, and valine, were therefore significantly higher under drought treatment. Histidine and valine were the most abundant essential amino acids in developing potato tubers (Figure 2A). The majority of non-essential amino acids had similar concentrations in developing tubers regardless of the treatment. Only glutamic acid showed a marked increase of 5.75 µmol g^−1^ under reduced soil moisture conditions. Concentrations of cysteine, proline, and serine were highest among all amino acids measured (Figure 2B).

### 3.3. Differential Gene Expression and Regulatory Cascades in Developing Tubers Under Drought Stress

**Figure 3:**
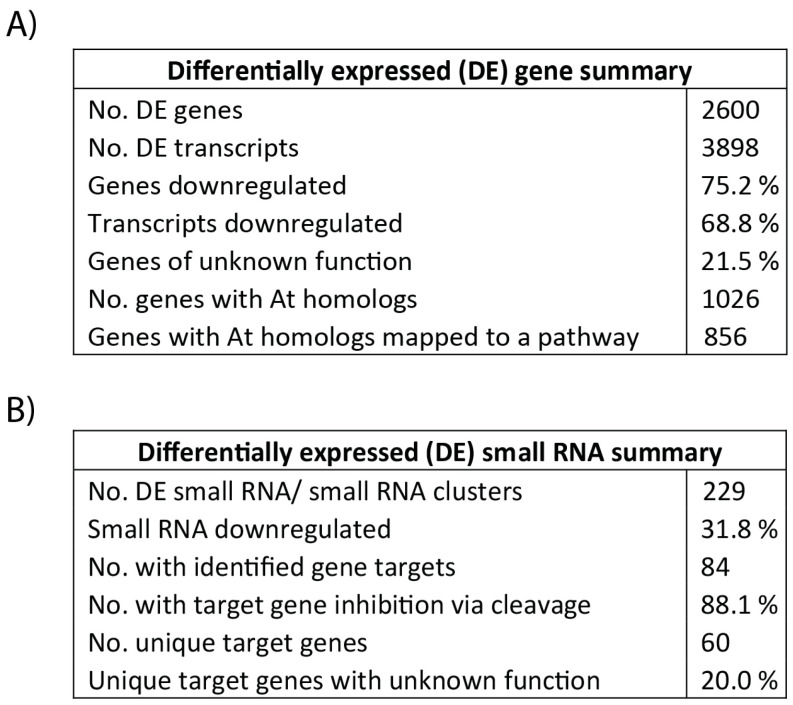
Summaries of differentially expressed genes (**A**) and small RNA (**B**) in potato tubers at 35% FC using a threshold of 4-fold difference in expression and a 5% FDR. Gene homologs in *A. thaliana* were considered if identity was greater than 50%. Functional annotation clustering to KEGG pathways was based on *Arabidopsis* gene IDs using the DAVID Bioinformatics online resource 6.8 (https://david.ncifcrf.gov/).

**Figure 4:**
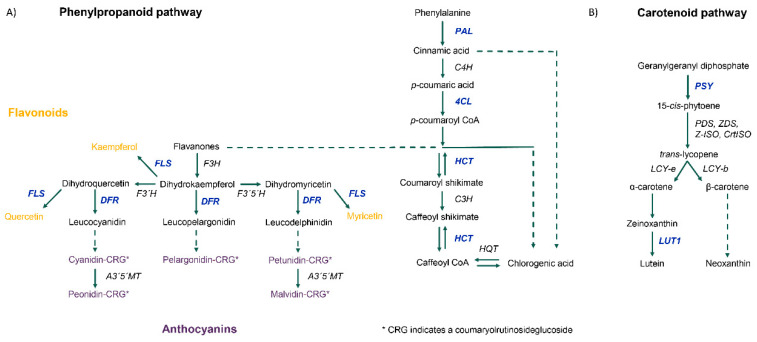
Diagrams depicting key enzymes in the phenylpropanoid (**A**) and carotenoid (**B**) biosynthetic pathways. Genes that are significantly down-regulated beyond cut-off values of 5% FDR and a log-fold change greater than 2 are written in blue.

Tubers subjected to soil moisture deficit showed both differential gene and transcript expression, with 75.2% and 68.8% being down-regulated, respectively. One-fifth of genes with differential expression were of unknown function. A full summary of the observed changes is listed in Figure 3A with a list of all differentially expressed genes provided in Supplementary Table S1. Down-regulated genes include those with functions in ABA, auxin and ethylene signaling as well as in auxin, carotenoid, and phenylpropanoid biosynthesis. Up-regulated genes have roles in amino acid biosynthesis, function as molecular chaperones and are involved in ubiquitin-driven proteolysis. Gene names, functional annotation, and the corresponding *Arabidopsis* homologs used for pathway mapping can be found in Table 2. Focusing on the regulation of the phenylpropanoid pathway, fifteen annotated MYB transcription factors were down-regulated by more than 4-fold under low-soil moisture conditions. Up-regulated transcription factors include two MYB transcription factors and one LIM transcription factor. No other MYB, KNOX or LIM transcription factors were above the cut-off values of 5% FDR and a log-fold change greater than two. Genes with key functions in the phenylpropanoid and carotenoid pathways that were down-regulated in potato tubers at 35% FC are highlighted in Figure 4. Raw read counts for gene expression analysis can be found in Supplementary Table S2.

The isolated small RNA were grouped into 87,213 clusters with an additional 10,209 unassigned sequences. Of these, 103 clusters and 126 unassigned sequences were differentially expressed. Additional summary statistics are listed in Figure 3B. Differentially expressed small RNA clusters with identified gene targets are listed in Supplementary Table S3. None of the small RNA clusters with target genes listed in Table 2 showed differential expression between the two treatments. Expression of target MYB transcription factor genes was also not correlated to small RNA cluster expression (Supplementary Table S4). Interestingly, the expression of small RNA clusters primarily targeting patatin genes was significantly up-regulated and negatively correlated to target gene expression (*r* = −0.61). These clusters and their targets can be found in Table 3.

## 4. Discussion

Optimal potato tuber growth occurs around 20 °C and plants are susceptible to losses in productivity under hot, arid conditions. Such conditions are expected to increase in the coming decade, therefore functional indicators of plant stress and the cascading effects on the developing tubers were evaluated. In this study, the Canadian potato cultivar “Vigor” was gradually exposed to increasing water deficit to a level of 35% FC beginning at the start of the tuber bulking phase (Figure 1). As seen previously in the literature, fluorescent measurements were not distinguishable between treatments [15] and thus were not dependable indicators of drought stress in potato plants (Table 1). At the plot level, there were no significant differences in yield or tuber number between the treatments (Table 1), however, discrepancies could become more prominent in commercial field production. Canopy temperature was considerably elevated in the drought treatments and has evidence supporting its use for drought stress assessments [30]. Average concentrations of ABA trended upwards in potato tubers exposed to water deficit compared to well-watered controls, suggesting drought responses had been initiated. Gene expression data further corroborated that drought signaling pathways had been activated as there was marked down-regulation of an ABA receptor PYR1, down-regulation of a series of small auxin up-regulated RNA (SAUR) genes involved in cell expansion and organ elongation in response to the environment [31] and the up-regulation of heat shock factor proteins [32] (Table 2). Notably, outside of common changes to the regulation of heat shock proteins, several genes previously identified as differentially expressed in severely drought-stressed potato leaves [32] were inversely regulated in the mildly stressed potato tubers collected in this study. These include the WRKY transcription factor (PGSC0003DMG400001434) and the developmental gene UPA16 (PGSC0003DMG400031742) [32] which had 2-fold and 78-fold increases in expression compared to well-watered control tubers. Lists of genes implicated in drought stress response in potato leaf tissue [33] and potato stolons [34] have been compiled and here we provide those in developing tubers (Table 2; Supplementary Table S1).

Metabolic effects of drought on parameters such as free amino acids, soluble protein, and phenolics were assessed. The gradual drought stress to which the potato tubers were exposed resulted in no significant differences in total free amino acid concentrations, although treatment averages appeared to be divergent with 154.1 and 207.2 µmol g^−1^ in the control and drought treatments, respectively. These differences could be associated with the up-regulation of genes involved in proteolysis (Table 2). Elevated concentrations of proline have been shown to indicate stress in potato leaves [35], however, similar concentrations were observed in tubers irrespective of treatment. The largest changes occurred in the amino acid profile, where concentrations of branched-chain amino acids leucine and isoleucine increased (Figure 2). This indicated a greater proportion of dietary essential amino acids. Increases in branched-chain amino acids are likely attributable to the up-regulation of acetolactate synthase (Table 2), which is the first enzyme in the branched-chain amino acid synthesis pathway [36].

A major fraction (up to 40%) of the soluble protein in potato tubers consists of glycoproteins, known as patatins, that act both as storage proteins and show activity as non-specific lipid acyl hydrolases (LAH) with potential roles in plant defense against biotic stressors [37,38]. In the case of abiotic stress, it was observed that five patatin genes were down-regulated by at least 16-fold with the regulation of gene expression likely occurring via an increased presence of small RNA (Table 3). A possible consequence is reduced protein content in the resulting potato tubers.

As one of the major sources of plant phenolics in the human diet, potatoes have been targeted in breeding for greater total phenolics and antioxidant capacity [3,39]. Phenolic content is known to show a high degree of environmental plasticity with cooler temperatures during the growing period and storage, attributed to higher average accumulation [4,5]. Under drought conditions, expressions of key enzymes required for the biosynthesis of anthocyanins (DFR), flavonoids (FLS) and chlorogenic acid (HCT) were drastically reduced (Figure 4A). Initial flow into the phenylpropanoid pathway through PAL was also reduced, leading to the accumulation of phenylalanine observed in Figure 2. Similar results have been previously observed in the literature [5]. Key enzymes of the carotenoid pathway were also downregulated (Figure 4B). Environmental conditions leading to the repression of phenolic biosynthesis could minimize gains achieved in breeding programs. Regulation of the phenylpropanoid pathway can occur via MYB transcription factors [8], fifteen of which were significantly suppressed under drought (Supplementary Table S1). Unlike previous findings in potato leaves [10], expressions of small RNA and their target MYB transcription factors were not correlated in the drought-stressed tubers. There was therefore no evidence to suggest that small RNA played a role in regulating the phenylpropanoid or carotenoid pathways under drought conditions in potato tubers (Supplementary Table S4).

## 5. Conclusions

Potato is among one of the most important food crops, yet maintaining plant productivity in this drought-sensitive crop has become a challenge. From a nutrition perspective, decreasing soil water availability during tuber filling, as a function of a warming climate or as a production practice to induce senescence for an earlier harvest, may lead to a reduction in tuber quality. While mild drought increases the proportion of essential amino acids, potential losses in protein and phenolic content would outweigh the benefit. While MYB transcription factors may be targeted to reduce effects on the phenylpropanoid pathway, identification of small RNA as the regulator of patatin gene expression suggests it may be difficult to maintain patatin expression in drought-susceptible cultivars using current breeding techniques.

## Figures and Tables

**Table 1:** Agronomic and physiological traits averaged for drought (35% field capacity) and control (70% field capacity) plots with standard error of the mean in parentheses. Bold numbers indicate significance (*p* < 0.05) between treatments.

Treatment	Yield (g)	No. of Tubers	CCI	Fv/Fm	Canopy Temp (°C)
35% FC	1381.7 (248.1)	12.8 (0.8)	19.5 (0.2)	0.24 (0.05)	**28.0 (0.3)**
70% FC	1737.3 (198.1)	18.1 (3.5)	15.4 (1.6)	0.38 (0.03)	24.1 (0.9)

**Table 2:** List of differentially expressed genes in drought-stressed potato tubers, the corresponding homologs in *A. thaliana,* and the pathway in which they participate. Pathway mapping was done based on the *Arabidopsis* gene names and similarities between the original *S. tuberosum* gene and its homolog are expressed as the percentage of identical base pairs in the gene sequences (% ID).

Gene Regulation	Pathway	Gene Name	Description	Log2 Fold Change	At Homologs	Descriptor	% ID
Down-	ABA signaling	PGSC0003DMG400002100	Abscisic acid receptor PYR1	−2.07	AT4G17,870	PYR1	72.2
regulated					AT5G46,790	PYL1	61.0
	Auxin	PGSC0003DMG400001589	Amino acid transporter	−5.30	AT2G21050	LAX2	86.3
	biosynthesis	PGSC0003DMG400024978	Indole-3-acetic acid-amido	−5.06	AT2G14960	GH3.1	77.3
	and signaling		synthetase GH3.3		AT2G23170	GH3.3	74.1
					AT4G37390	GH3.2	73.3
					AT1G59500	GH3.4	69.8
		PGSC0003DMG400024997	Indole-3-acetic acid-amido synthetase GH3.6	−2.17	AT5G54510	GH3.6	70.8
		PGSC0003DMG400014707	Flavin monooxygenase	−3.42	AT4G28720	YUC8	68.3
					AT5G43890	YUC5	67.2
		PGSC0003DMG400026087	Flavin monooxygenase	−3.09	AT5G11320	YUC4	57.4
					AT4G32540	YUC	54.3
		PGSC0003DMG400003773	SAUR family protein	−8.34	AT1G75580	SAUR51	72.2
					AT1G19830	SAUR54	61.5
		PGSC0003DMG400001667	SAUR family protein	−7.40	AT4G38860	SAUR16	64.8
					AT4G34760	SAUR50	64.5
					AT2G21220	SAUR12	63.5
					AT2G16580	SAUR8	63.0
		PGSC0003DMG400001614	SAUR family protein	−3.75	AT4G34760	SAUR50	75.7
					AT4G38860	SAUR16	73.3
					AT2G16580	SAUR8	71.3
					AT2G21220	SAUR12	71.1
		PGSC0003DMG400001668	SAUR family protein	−3.71	AT4G38860	SAUR16	77.1
					AT4G34760	SAUR50	76.6
					AT2G21220	SAUR12	75.0
					AT2G16580	SAUR8	70.4
		PGSC0003DMG400001655	SAUR family protein	−2.98	AT4G34750	SAUR49	54.0
		PGSC0003DMG400022233	SAUR family protein ARG7	−2.93	AT3G12830	SAUR72	64.4
					AT1G16510	SAUR41	55.1
		PGSC0003DMG400001615	SAUR family protein	−2.06	AT4G34760	SAUR50	73.8
					AT4G38860	SAUR16	71.4
					AT2G21220	SAUR12	69.2
					AT2G16580	SAUR8	68.5
	Carotenoid biosynthesis	PGSC0003DMG400028180	Cytochrome P450-type monooxygenase 97C11	−2.07	AT3G53130	LUT1	77.2
		PGSC0003DMG400024063	Phytoene synthase 1, chloroplastic	−5.07	AT5G17230	PSY	64.3
	Ethylene signaling	PGSC0003DMG400014204	Transcription factor TSRF1	−3.57	AT3G23240	ERF1	51.4
	Phenylpropanoid	PGSC0003DMG400003605	Dihydroflavonol 4-reductase	−5.19	AT5G42800	DFR	59.2
	biosynthesis	PGSC0003DMG400014093	Flavonol synthase	−2.19	AT5G08640	FLS1	62.5
					AT5G63590	FLS3	50.3
		PGSC0003DMG400014152	Hydroxycinnamoyl transferase	−2.00	AT5G48930	HCT	77.8
		PGSC0003DMG400023458	Phenylalanine ammonia-	−4.68	AT3G10340	PAL4	79.9
			lyase		AT5G04230	PAL3	73.2
		PGSC0003DMG400014223	4-coumarate--CoA ligase 2	−2.30	AT3G21240	4CL2	68.5
					AT1G51680	4CL1	67.9
					AT3G21230	4CL4	58.9
		PGSC0003DMG400028929	4-coumarate--CoA ligase 2	−2.00	AT3G21240	4CL2	69.2
					AT1G51680	4CL1	68.8
					AT3G21230	4CL4	59.8
Up-regulated	Amino acid biosynthesis	PGSC0003DMG400034102	Acetolactate synthase	2.20	AT3G48560	CSR1	76.9
	Protein folding	PGSC0003DMG400008223	Heat shock factor protein HSF30	4.44	AT2G26150	HSFA2	51.0
		PGSC0003DMG400003219	Small heat shock protein, chloroplastic	4.11	AT4G27670	Heat shock protein 21	53.7
		PGSC0003DMG400030341	Small heat shock protein-Class I 17.6kD	3.99	AT2G29500	HSP17.6B	77.8
		PGSC0003DMG400024707	Small heat shock protein	2.90	AT1G09080	Heat shock protein 70	75.1
		PGSC0003DMG402028907	Small heat shock protein 90	2.72	AT5G52640	Heat shock protein 90	52.0
		PGSC0003DMG400030426	Small heat shock protein-Class I 17.6kD	2.50	AT2G29500	HSP17.6B	74.5
	Proteolysis	PGSC0003DMG400006185	Skp1 1	2.56	AT1G75950	SKP1	74.4
		PGSC0003DMG400006184	Skp1	2.20	AT1G75950	SKP1	75.0

**Table 3:** List of differentially expressed small RNA clusters in drought-stressed potato tubers that negatively correlate to target transcript expression. Target alignments, gene ID, expression and descriptions are included.

Small RNA Cluster	Log2 Fold Change	Target Alignment	Target Gene	Log2-Fold Change	Protein Description
Cluster 34023	5.03	AGCUCAUUAAUCUCUUCGAUA	PGSC0003DMG400009921	−6.24	Cysteine protease 14
Cluster 23921	4.68	AGGGUUCAAGAAAAUGCAUUA	PGSC0003DMG400029247	−4.75	Patatin group O
Cluster 15144	4.62	AGGGUUCAAGAAAAUGCAUUA			
Cluster 41775	4.49	ACCUCAGGGUUCAAGAAAAUG			
Cluster 83189	5.49	AGGCACUGGCACUACUUCAGA	PGSC0003DMG400017091	−4.25	Patatin-01; Probable lipolytic
Cluster 83175	4.98	AGCCAGUAAUAUUCACCAAGU			acyl hydrolase
Cluster 83174	3.45	AGGCACUGGCACUACUUCAGA			
Cluster 7920	4.95	GGCAGCAAGUUCUUACAUGAC	PGSC0003DMG400008749	−4.06	Patatin-05; Probable lipolytic
Cluster 68384	3.01	AUCAUUCCGGGUAUCAUUCUC			acyl hydrolase
Cluster 83190	2.87	UUCCGGGUAUCAUUCUCGAAU			
Cluster 83166	2.66	UCCGGGUAUCAUUCUCGAAU			
Cluster 68380	5.49	AGGCACUGGCACUAAUUCAGA	PGSC0003DMG400014104	−4.47	Patatin-2-Kuras 4; Probable
Cluster 83164	5.49	AGGCAGCUAAAUGGGGUCCUC			lipolytic acyl hydrolase
Cluster 20497	5.38	CUGUUGGUGAUCCGGCGUUA			
Cluster 68397	5.36	GUUGCUACUGUUGGUGAUCCG			
Cluster 83182	4.97	GGCACUACUUCAGAGUUUGAU	PGSC0003DMG401017090	−4.91	Patatin-3-Kuras 1

## Supplementary Materials

The following are available online at https://www.mdpi.com/2073-4425/11/8/864/s1; Figure S1: Experimental conditions; Table S1: DE genes; Table S2: Raw read counts; Table S3: DE small RNA; Table S4: small RNA, targets expression.
