# Supplementary figures and images for: Drought-Induced Regulatory Cascades and Their Effects on the Nutritional Quality of Developing Potato Tubers
Source: Genes (Basel). 2020 Jul 30;11(8):864. doi: 10.3390/genes11080864 (PMC7465940; doi:10.3390/genes11080864)

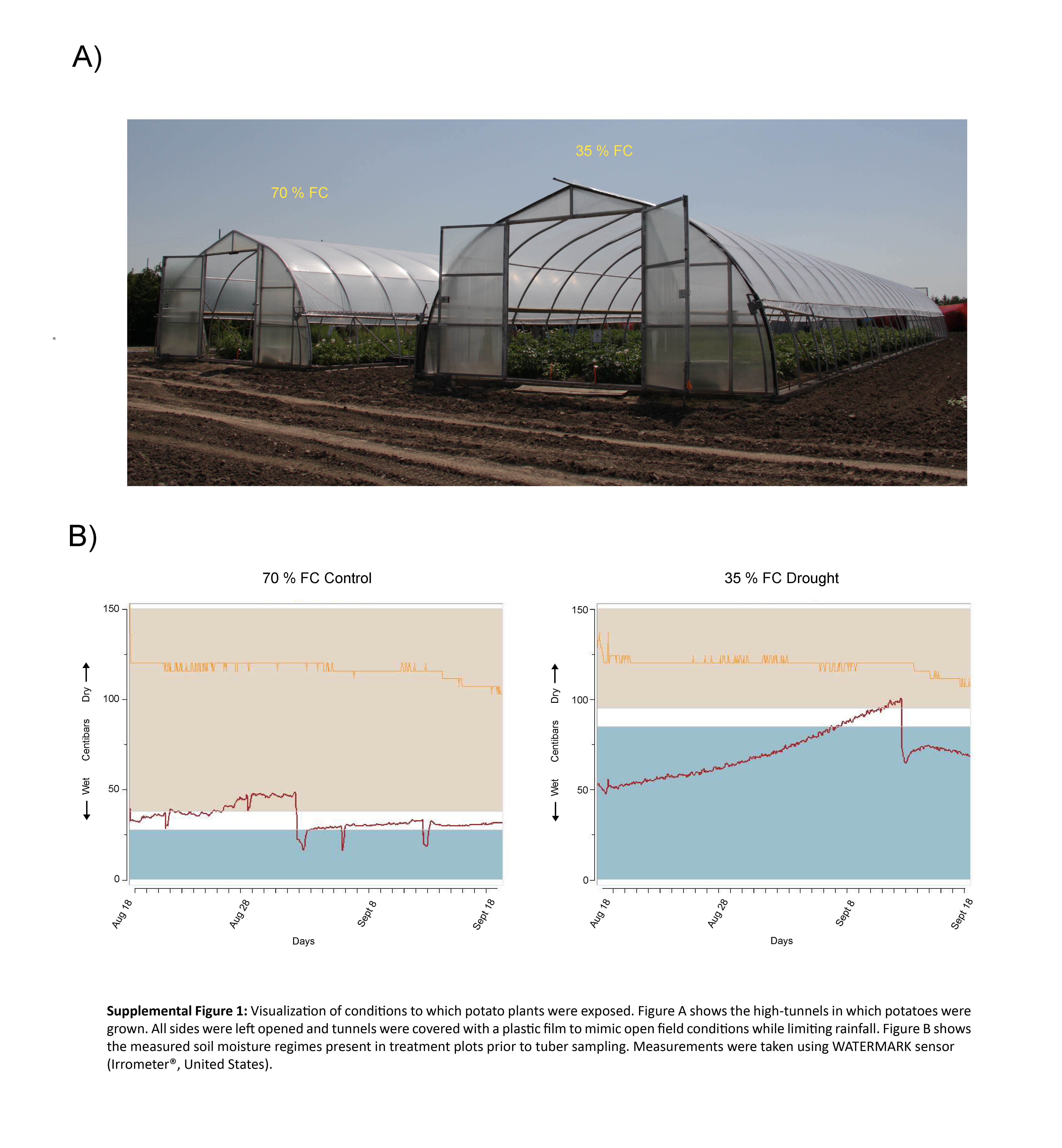

Supplement: Supplementary file 1 [file genes-11-00864-s001.zip › Supplementary Results/FigS1_ExperimentalConditions.jpg]
